# Triple null mutations in starch synthase *SSIIa* gene homoeologs lead to high amylose and resistant starch in hexaploid wheat

**DOI:** 10.1186/s12870-020-02822-5

**Published:** 2021-02-03

**Authors:** Adam Schoen, Anupama Joshi, Vijay Tiwari, Bikram S. Gill, Nidhi Rawat

**Affiliations:** 1 Department of Plant Science and Landscape Architecture, University of Maryland, College Park, MD 20742 USA; 2 Department of Plant Pathology, Kansas State University, Manhattan, KS 66506 USA

**Keywords:** Resistant starch, Starch synthase, Amylose, TILLING, Null mutations, Health benefits

## Abstract

**Background:**

Lack of nutritionally appropriate foods is one of the leading causes of obesity in the US and worldwide. Wheat (*Triticum aestivum*) provides 20% of the calories consumed daily across the globe. The nutrients in the wheat grain come primarily from the starch composed of amylose and amylopectin. Resistant starch content, which is known to have significant human health benefits, can be increased by modifying starch synthesis pathways. Starch synthase enzyme *SSIIa,* also known as starch granule protein isoform-1 (*SGP-1*), is integral to the biosynthesis of the branched and readily digestible glucose polymer amylopectin. The goal of this work was to develop a triple null mutant genotype for *SSIIa* locus in the elite hard red winter wheat variety ‘Jagger’ and evaluate the effect of the knock-out mutations on resistant starch content in grains with respect to wild type.

**Results:**

Knock-out mutations in *SSIIa* in the three genomes of wheat variety ‘Jagger’ were identified using TILLING. Subsequently, these loss-of function mutations on A, B, and D genomes were combined by crossing to generate a triple knockout mutant genotype Jag-*ssiia*-∆ABD. The Jag-*ssiia*-∆ABD had an amylose content of 35.70% compared to 31.15% in Jagger, leading to ~ 118% increase in resistant starch in the Jag-*ssiia*-∆ABD genotype of Jagger wheat. The single individual genome mutations also had various effects on starch composition.

**Conclusions:**

Our full null Jag-*ssiia*-∆ABD mutant showed a significant increase in RS without the shriveled grain phenotype seen in other *ssiia* knockouts in elite wheat cultivars. Moreover, this study shows the potential for developing nutritionally improved foods in a non-GM approach. Since all the mutants have been developed in an elite wheat cultivar, their adoption in production and supply will be feasible in future.

**Supplementary Information:**

The online version contains supplementary material available at 10.1186/s12870-020-02822-5.

## Background

Around 13% of the people in the world are obese and according to available data, obesity is one of the largest risk factors for human deaths globally [1]. Diseases caused by poor diet, such as cardiovascular issues and type-2 diabetes, are directly related to obesity [1, 2]. In 2017, roughly 8% of deaths worldwide were a result of obesity related diseases [1]. Obesity is an effect of a sedentary lifestyle and an overconsumption of cheap, high caloric foods [2]. In order to get a better handle on an epidemic of this magnitude, developing affordable food sources with higher nutrition are imperative. Wheat, on average, is responsible for over 500 cal consumed daily worldwide, which equates to roughly 20% of the recommended daily calories [3, 4]. Enhancing the nutritional value of such an important food source can be a powerful tool in fighting obesity.

The calories and nutrition that are associated with wheat products come primarily from starch, which serves as the energy and carbon reservoir for the embryo. Roughly 70–90% of the dry weight of the endosperm is composed of starch [5, 6]. Starch is made up primarily of alternating sections of glucose polymers; linear amylose and highly branched amylopectin [6, 7]. Unbranched chains are organized by α-(1,4)-linked glucose, whereas branched sections are composed of α-(1,4)-linked glucose as well as branched α-(1,6)-linked glucose [8, 9]. Regions of highly branched amylopectin alternate with linear arrays of double helices that contribute to the semi-crystalline composition of starch granules [10]. Starches, as a whole, are categorized based on their digestibility: Rapidly digestible starches (RDS) and slowly digestible starches (SDS). RDS are converted into their glucose constituents within the first 20 min of incubation with starch degrading enzymes. The SDS are completely digested in the small intestine. The resistant starches (RS) are the small fraction of starches that are resistant to α-amylase activity and are fermented by colonic bacteria [11, 12].

Studies on RS have shown incredible benefits to human health ranging from maintaining healthy bowel function to helping moderate glycemic indices, as well as the possibility to prevent colon cancer [11–14]. RS intake is lower in populations where obesity related diseases are prevelent [15] .Replacement of RDS with RS, has been shown to lower the rate of glucose entry into the blood stream, thus resulting in a reduction of insulin demand [14]. In rats, fat tissue was reduced in individuals fed with a high RS diet [16]. In an empirical study done by Regina et al. (2006), rats fed with a novel high RS transgenic wheat were found to have a roughly 100% increase in short chain fatty acid (SCFA) pools in large bowel digesta, as well as fecal excretion. Additionally, a lower pH was recorded in the bowels of these rats, indicating colonic fermentation [15]. SCFAs have been shown to increase colonic blood flow, as well as lower the risk of malignant transformation. In addition, SCFAs play a role in acidifying digesta content, which has the ability to inactivate toxic compounds [14, 17, 18]. Furthermore, the obesity epidemic in the U.S., as well as world-wide, has a direct impact on type 2 diabetes and/or cardiovascular disease [2, 19]. In comparison with diets high in digestible starch (DS), diets consisting of higher RS showed a decrease in adipocyte cell size, as well as a reduced whole-body weight gain in rats [16].. In humans, Park et al. (2004) showed evidence that RS dietary supplementation showed a decrease in blood cholesterol concentrations [20]. Moreover, studies have also shown longer times of satiety after eating foods containing high RS [21, 22]. In terms of helping those who have already developed type 2 diabetes, human study showed a significant decrease in insulin levels after subjects ingested bread with higher RS content [23]. In order to develop wheat with higher RS, the ratio of amylose to amylopectin needs to be modified. Studies have shown that starches with higher amylose content are more resistant to digestion than those higher in amylopectin [18, 24].

Starch synthesis within the endosperm of wheat is accomplished by several enzymes, with differing roles in the elongation and branching of glucose polymers. Granule-bound starch synthase (GBSS) isoforms are the sole enzymes responsible for synthesis of amylose in starch formation [25]. Amylose-free, also refered to as waxy wheats, have been created through mutagenesis as well as crosses of cultivars lacking functional copies of GBSS homoeologs [26, 27]. Amylopectin synthesis is more complicated due to the complex, yet organized structure of the branching pattern, and requires more enzymes in the biosynthesis pathway. Starch branching enzymes (SBEs) and debranching enzymes (DBEs) work together to create the α-(1,6) branching patterns that are characteristic of amylopectin [10, 25]. Mutations in SBE genes in cereals, including durum (*Triticum durum*) (genome AABB) and bread wheat (genome AABBDD), have shown an increase in amylose [7, 28, 29]. Starch synthase enzymes (SS) are responsible for the short chains of glucose polymers between branched clusters, thus are important for the organization of the higher structure of starch granules [10, 25, 30]. Individuals with full null SS genes in bread wheat have shown increases in amylose and RS content [31, 32]. Studies in durum and bread wheat have shown similar results, however the grains of these individuals were highly shriveled [7, 33–35].

In this study, an EMS (ethyl methanesulfonate) mutagenized TILLING (Targeting Induced Local Lesions IN Genomes) population of hard red winter bread wheat variety ‘Jagger’ was used to study the impact of knock-out mutations of starch synthase genes on the RS and amylose content [36]. Jagger was selected for this study due to its superior agronomic performance and excellent baking properties [37]. TILLING is a powerful forward and reverse genetics tool where chemically or radiation induced SNPs are identified via high throughput screening methods [36, 38–42]. Several genes have been validated using TILLING, including genes that are responsible for starch synthesis [34, 35, 38, 42]. With the increasing amount of reference genomes available, as well the decreasing price for sequencing techniques, TILLING has become a popular functional genetics tool. In addition, development of cultivars with modified genes using TILLING techniques is not considered a GM approach and is not subject to the public and legal stigmas that surround GM crops.

With the use of our Jagger TILLING population, knockout mutants of *SSIIa* gene homoeologs were identified in “A”, “B”, and “D” sub genomes and combined by crossing to develop a full null *ssiia* mutant. With increased RS content in these individuals, we show the ability to develop increased nutritional value in an important cereal crop without the use of transgenics.

## Results

### Development of jag-*ssiia*-∆ABD mutant

Two predictive knock-out mutants each were found for B and D genomes, whereas only one was found for A genome (W544*; Table 1). The mutations leading to earlier truncation of the proteins were selected for the B (Q601*) and D (W544*) genomes for developing the full null mutant. It is interesting to note that the A and D genome truncation mutant were at the same position of protein coded by the different homoeoalleles. A double cross strategy was used to combine mutations in the three genomes while maintaining (B genome) mutation in homozygous condition. Six were found to have homozygous mutations in all the three genomes out of 96 plants from the selfed progeny, which fits the expected genetic segregation of 15:1. The homozygous full null mutant, mutants of A genome (W544*), B genome (Q601*) and D genome (W544*) along with wild type parent Jagger were used to study starch composition parameters. The sequences of A, B, and D genome amplicons targeted for TILLING from wild type Jagger and knock-out mutants and their predicted protein sequences have been provided in Supplementary Table 1 and 2, respectively.

**Table 1:** List of primers, amplicon sizes, number of individuals covered by TILLING, number of mutants obtained, and the knock-out mutants obtained for the three homoeoalleles of *SSIIa*. Entries in bold indicate knock-out mutants selected for developing full null mutants, and for starch composition analyses

Primer name	NCBI Accession of the gene (Shimbata et al. 2005)	Genome	5′-3′ Sequence	Product Size	Number of M2 Individuals covered	Total number of Mutants obtained	Knock-out Mutant Identity	Nucleotide change	Position of truncation in the knock-out mutants
SSIIa-AF	AB201445	A	TTCCTCTATAATGATCACATGC	1050	1032	46	**Box-10-H8**	**G > A**	W544*
SSIIa-BF	AB201446	B	GAATTAGTACATGCTTTGGTCGC	1042	984	48	**Box-2-F4** Box-13-C5	**C > T** G > A	Q601* W762*
SSIIa-DF	AB201447	D	TATACAACACTGACATGCCGAA	1052	1260	52	**Box-2-G5** Box-7-E5	**G > A** G > A	W544* W555*
SSIIa-R	–	Common	TCACCACTGGTACTTGGCCTTG	–	–	–	–	–	–

* denotes truncation of the protein

### Grain size and weight

**Fig. 1:**
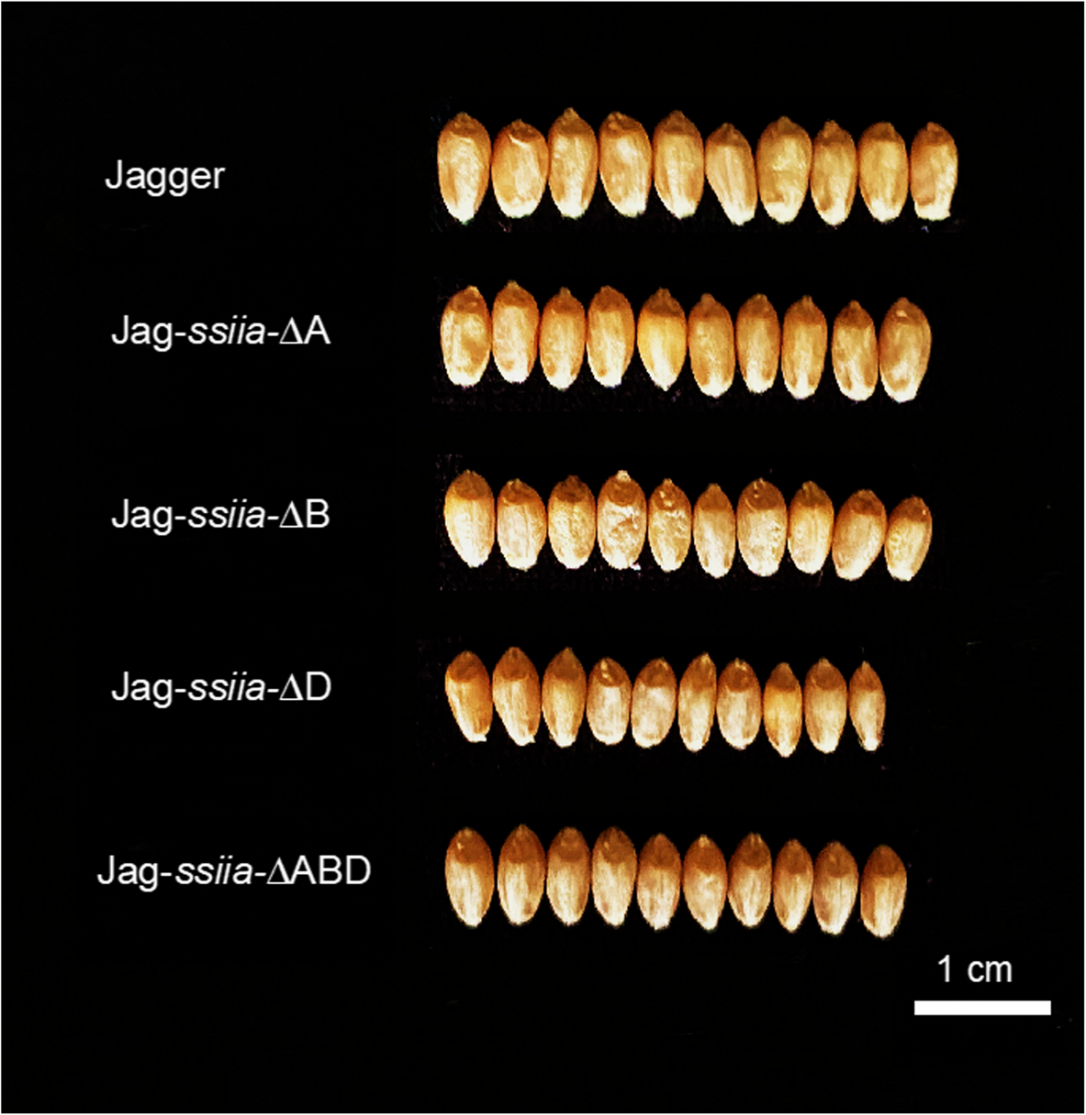
Side-by-side comparison of ten grains each of the different genotypes to portray the differences in grain width

Grain width differed between Jag-*ssiia*-∆ABD and WT (*P* < 0.01) genotypes. Interestingly, Jag-*ssiia*-∆B seeds were widest among all the mutant combinations studied, although they did not show any significant difference in grain width in comparison with WT. Between genotypes, Jag-*ssiia*-∆B showed a significant increase (*P* < 0.0001) in grain width in comparison with Jag-*ssiia*-∆A, *ssiia* ∆ D, and Jag-*ssiia*-∆ABD (Fig. 1). The ANOVA test for grain length showed no significant (*P* = 0.8) difference between the genotypes (Table 2).

**Table 2:** Resistant starch content, total starch content, amylose and grain size parameters of mutant and WT Jagger grains

Genotype	Resistant starch	Total starch	Amylose	Grain width	Grain length	TGW
	(%)	(g/100 g)	(%)	(mm)	(mm)	(g)
Jagger	1.01 ± 0.13^a^	54.24 ± 1.58^d^	31.15 ± 0.84^a^	3.48 ± 0.03^b^	7.07 ± 0.05	41.59 ± 0.47^c^
Jag-*ssiia*-∆A	1.42 ± 0.11^a^	44.42 ± 0.88^ab^	32.5 ± 0.96^ab^	3.20 ± 0.06^a^	6.97 ± 0.1	32.7 ± 0.74^a^
Jag-*ssiia*-∆B	0.98 ± 0.1^a^	48.5 ± 0.90^ac^	30.75 ± 0.73^a^	3.59 ± 0.05^b^	7.06 ± 0.09	36.07 ± 0.54^b^
Jag-*ssiia*-∆D	1.19 ± 0.1^a^	51.03 ± 0.94^cd^	27.47 ± 0.65^c^	3.20 ± 0.06^a^	7.01 ± 0.08	33.15 ± 0.57^a^
Jag-*ssiia*-∆ABD	2.21 ± 0.07^b^	44.96 ± 0.67^b^	35.70 ± 1.07^b^	3.21 ± 0.06^a^	6.95 ± 0.06	32.73 ± 0.64^a^

Different letter superscripts indicate significant differences between means for *P* < 0.05

In thousand grain weight (TGW), a significant decrease was observed between Jag-*ssiia*-∆ABD and WT (*P* < 0.00001). Similarly, all other genotypes showed a significant decrease (*P* < 0.00001) in TGW in comparison with WT as well. A 21.29% decrease in TGW on average was observed between WT and Jag-*ssiia*-∆ABD. Surprisingly, Jag-*ssiia*-∆B had a slightly, but significantly (*P* < 0.01) higher TGW than Jag-*ssiia*-∆A, *ssiia* ∆ D, and Jag-*ssiia*-∆ABD, which is consistent with grain width data.

### Amylose, RS, and Total starch content

**Fig. 2:**
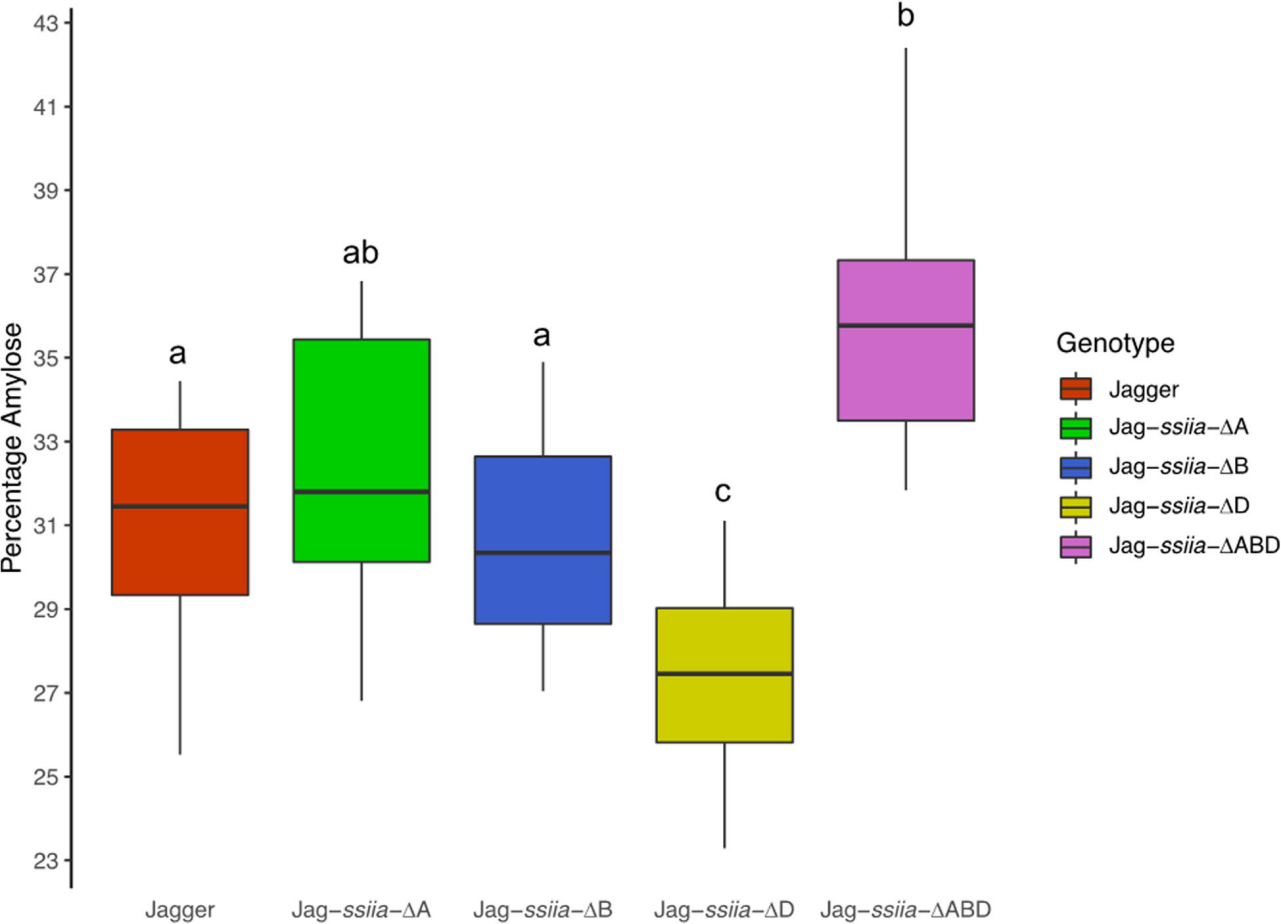
Percentage of amylose quantified in flour derived from mutant grains. Different letters signify significant differences between means (*P* < 0.05)

The results of the amylose/amylopectin assay showed a significant increase in amylose in Jag-*ssiia*-∆ABD in comparison with WT (P < 0.01), with WT having an amylose content of 31.15% on average, and 35.70% on average in Jag-*ssiia*-∆ABD. Interestingly, Jag-*ssiia*-∆D had a significant decrease in amylose content in comparison to WT (*P* < 0.001). Similarly, Jag-*ssiia*-∆ABD had a significant decrease in total starch (TS) (*P* < 0.0001) in comparison with WT. Additionally, Jag-*ssiia*-∆D showed no significant difference in total starch in comparison to WT. However a significant increase (*P* < 0.0001) in TS was observed between Jag-*ssiia*-∆D and Jag-*ssiia*-∆ABD (Fig. 2).

**Fig. 3:**
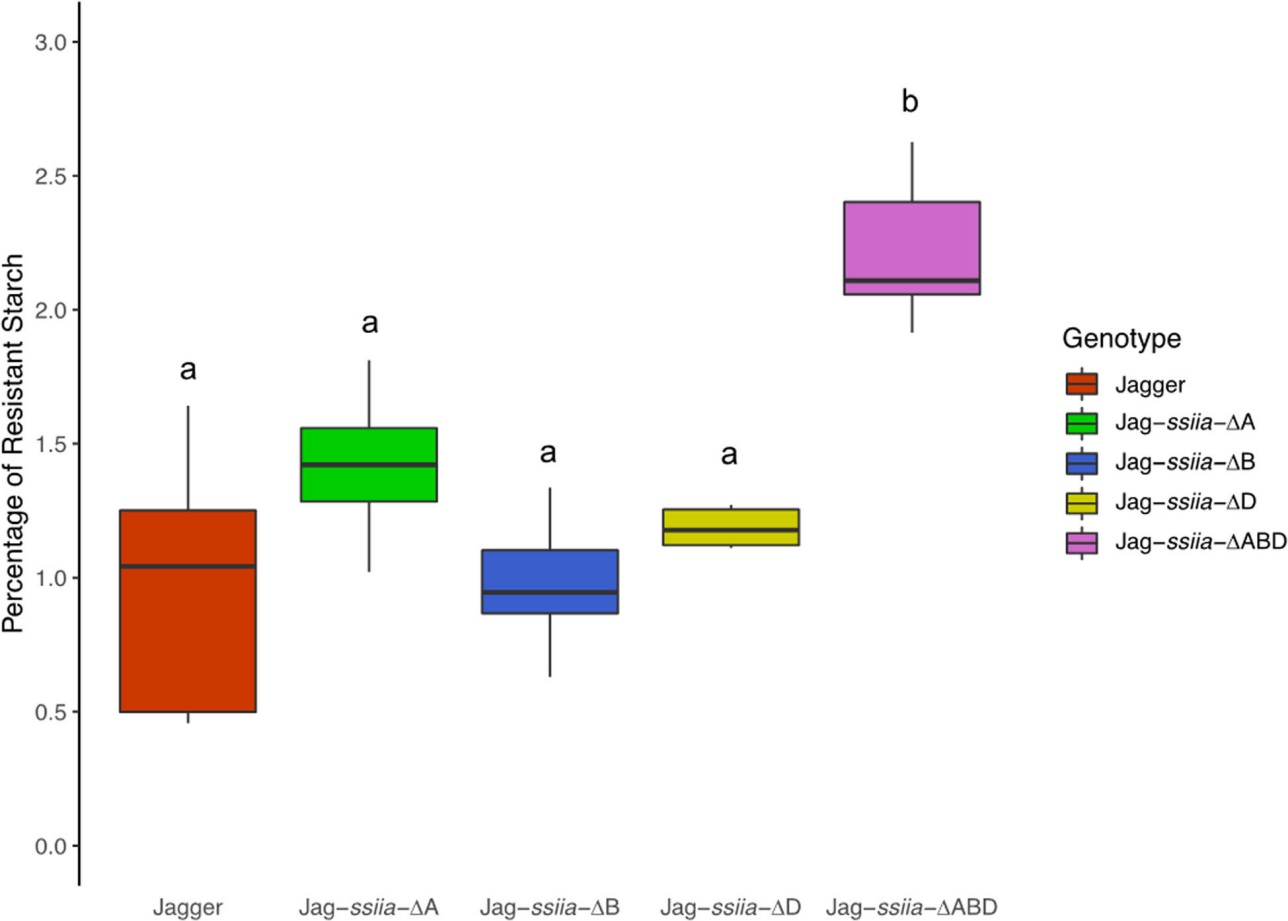
Resistant starch as a percentage total starch. Percentage of RS was calculated by dividing g/100 g RS by g/100 g TS. Different letters signify significant differences between means (*P* < 0.05)

The Jag-*ssiia*-∆ABD genotype had a significant increase in percent RS in terms of percent of total starch in comparison with WT (*P* < 0.0001), a total of a 118.81% increase in RS. Significant increases (*P* < 0.001) of RS percent were also observed between Jag-*ssiia*-∆ABD and every other genotype (Fig. 3). In terms of RS content (g/100 g), a significant increase in RS was observed in Jag-*ssiia*-∆ABD in comparison with WT (*P* < 0.0001). Between genotypes, Jag-*ssiia*-∆ABD was shown to have a significant increase (*P* < 0.01) in RS content in comparison with the other mutant genotypes (Table 2).

**Fig. 4:**
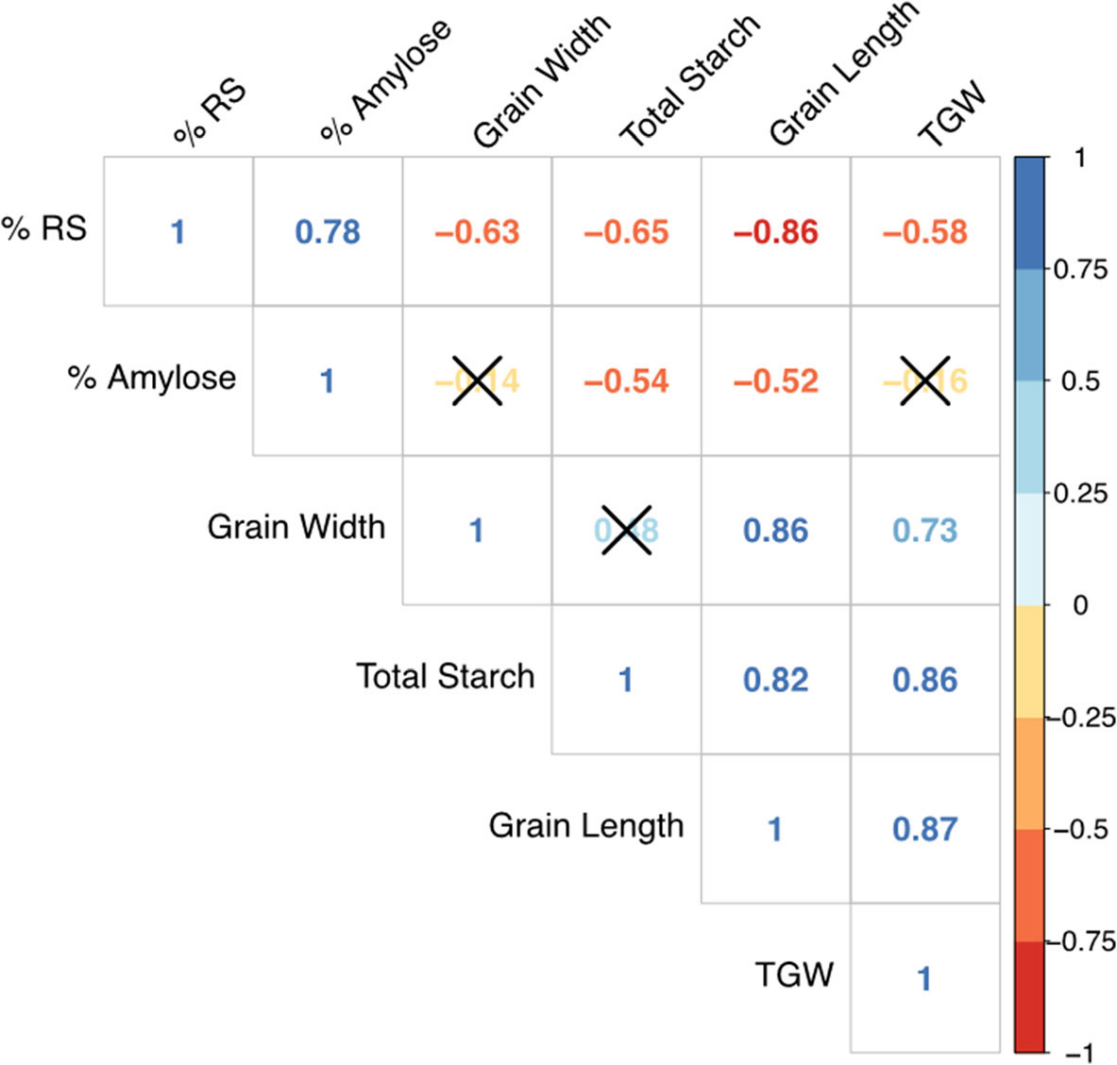
Correlation matrix for the different attributes of the mutant grains. Pearson correlations coefficients are displayed as colored numbers that show direction and strength of the correlations. Ellipses with ‘X’s’ denote insignificant correlations (*P* > 0.01)

A strong positive correlation (*r* = 0.78) was observed between percentage RS and amylose content. However, a moderate negative correlation was observed between percentage RS and TS (*r* = − 0.68). RS content (g/100 g) followed a similar pattern as percentage of resistant starch, but to a lesser degree. A strong positive correlation (*r* = 0.76) was observed between RS content and amylose content, and a moderate negative correlation (*r* = − 0.55) was observed between RS content and TS. A moderate negative correlation (*r* = − 0.63) was observed between amylose content and TS Fig. 4).

Moderately negative correlations were observed between percentage RS and grain width (*r* = − 0.63), as well as between percentage RS and TGW (*r* = − 0.58). A strong negative correlation was observed between percentage RS and grain length (*r* = − 0.86). TS and grain length showed a strong positive correlation (*r* = 0.82). Similar correlations were observed between RS content (g/100 g) and grain width (*r* = − 0.64), grain length (*r* = − 0.81), and TGW (*r* = − 0.47). Interestingly, amylose content showed no significant correlation (*P* > 0.01) with grain width. A weak positive correlation was found between TS and grain width (*r* = 0.46), and a strong positive correlation was found between total starch and TGW (*r* = 0.80).

## Discussion

Previous studies have shown the effects of null SBE and SS genes on starch composition in durum and hexaploid wheat backgrounds [7, 28, 29, 31, 32, 35, 42, 43]. Yamamori et al. (2000) developed a full null *ssiia* individual by crossing three different wheat varieties that contained null *ssiia* in each subgenome [31]. The full null *ssiia* genotype showed an increase in amylose content, as well as RS, however the seeds were highly shriveled [32]. Similar to this study, Botticella et al. (2018) developed a full null *ssiia* mutant in spring wheat cultivar ‘Cadenza’, named Cad-SSIIa*, which also showed increased amylose content and RS, however these grains were also highly shriveled.

In this study, we have developed a full null *ssiia* mutant, all within the genetic background of the elite cultivar ‘Jagger’. The full null mutant showed increases in RS and amylose content, similar to those reported for *ssiia* null mutants in durum wheat, as well as hexaploid wheat [30–32, 34]. Interestingly, our study showed variations within knock-out mutants of different genomes in relation to total starch and amylose content, suggesting a non-balanced homoeolog expression of *SSIIa* from the three genomes. It is known that ~ 30% of wheat genes show a non-balanced expression among the A, B and D genomes [36]. The Jag-*ssiia*-∆D mutant showed a decrease in amylose, in comparison with WT, and significantly more total starch than the Jag*-ssiia-*∆ABD mutant. The trend in Jag-*ssiia*-∆D is consistent with the expectation, as moderate negative correlations were observed between amylose content and total starch In Yamamori et al. (2000), cv Turkey, which naturally lacked the *ssiia* gene in the D genome, was used to integrate a null D genome *ssiia* gene into a full null individual [22]. No significant difference in amylose content between Turkey and Chinese Spring, and a slight increase between Turkey and Norin 61 was found, using a colorimetric as well as a titration assay, which were different than the protocols used in the present study. Chinese Spring and Norin 61 both contain functional *SSIIa* genes in all three genomes and were used as controls [31]. Non-balanced homoeolog expression of the three *SSIIa* homoeoalleles in Jagger or background mutations in the D genome mutant could be the underlying factors for the reverse effect on amylose content and TS of the *ssiia* D genome knock-out mutant. Further studies will be needed to identify the exact reason for this unexpected trend with the D genome copy of *ssiia*.

In studies of *ssiia* null mutant durum wheat, which is generally used for pasta flour, studies have shown a decrease in cooking time, increased firmness, and a resistant to overcooking in pasta. This, in addition to a higher protein and fat content are indicators of beneficial properties of flour derived from *ssiia* mutant wheat [33]. In addition, studies using high amylose bread wheat showed possibilities of using this flour as a replacement for both pasta flour as well as in steamed Chinese foods [44]. However, as this study shows, there are negative effects on yield-related traits such as TGW and GW in *ssiia* null mutants. Field-based experiments will be done in future once the background has been cleared of additional mutations by back-crossing to see if a detectable reduction in yield is observable due to exclusively the *ssiia* mutations. Additionally, some cooking properties have been shown to be negatively affected by high amylose content. Studies focusing on SBE genes in in durum and bread wheat have shown significant increases of 55.13 and 108.77% on average respectively in amylose content [5, 15, 42, 45]. Alternatively, studies on SS genes in durum and bread wheat have shown an amylose content increase of 62.74 and 17.90% on average respectively [30, 31, 33]. Morita et al. (2002) showed that high amylose bread wheat flour was unsuitable for bread making, as it resulted in dense, small pocketed loaves [37]. In an association study between 12 different soft wheat cultivars, Gaines et al. (2000) found a moderately negative correlation (*P* = − 0.53) between amylose content and milling flour yield [46]. However, it is important to note that the Jag*-ssiia-*∆ABD mutant can be used as a specialty wheat for targeting weight management and other health benefits in humans, however additional studies on non-starch polysaccharide (NSP) and sugar content may give further insight on the nutritional benefits of these grains.

The resistant starch content in our Jag-*ssiia*-∆ABD mutant was 118.81% higher than WT, which is significantly lower than studies on SBE gene knockouts. On average, SBE null mutants had an RS increase of roughly 652.95% in durum wheat, and 1132.39% in bread wheat [42, 45, 47]. This significant increase could be a result of the functionality of SBE genes. SBE genes are directly responsible for the branching of glucose polymers, whereas SS genes work on the linear portions of amylopectin [10, 25]. In durum wheat, a *ssiia* null individuals were developed by Botticella et al. (2016), and showed an increase in RS of 645% on average [30]. It is important to note, however, that these null *ssiia* mutants were developed by crossing two different varieties that had null *ssiia* genes in the A and B sub genomes and were highly shrivelled.

This study marks the first development of a full null *SSIIa* mutant in a single winter wheat background. It further exemplifies the power behind TILLING as a genetic tool. The benefits with working with a hexaploid wheat TILLING population are that a significantly smaller population of mutant individuals (< 2000 individuals) are required to get a knockout of any gene of interest. Tetraploid and diploid wheat species require significantly larger populations to have the same effect, ~ 3000 and ~ 5500 individuals, respectively [39, 40]. We used a previously established TILLING population, developed by Rawat et al. [36] and the full null mutants were all discovered in this population. Due to this individual being created via a TILLING approach, it circumvents the stigmas surrounding GM crops. The *ssiia* null individuals harbor several background mutations, but these can be significantly reduced through two to three rounds of back-crossing with the use of marker assisted breeding.

## Conclusions

A triple knockout mutant Jag-*ssiia*-∆ABD of an important gene in the starch synthesis pathway was developed in this study. The starch composition is similar to that of full null mutants in tetraploid backgrounds created through traditional breeding. In addition, seeds of Jag-*ssiia*-∆ABD genotype, though having a significant loss in TGW, still do not show a shriveled phenotype unlike previous hexaploid and durum *SSIIa* mutants. Subsequent experiments involving starch granule and overall grain morphology may give insight on why this is the case. The Jagger *ssiia* mutants are all in a single elite genetic background, making the phenotype less affected by compounding genes from different genetic backgrounds. However, it is important to note that the predictive combined mutants have not been back-crossed so far, leading to several background mutations not present in parental WT Jagger. The Jagger *ssiia* null mutants showed a significant increase in RS, a nutritionally important trait in cereals that has shown several health benefits in humans. Though the increase of RS was relatively small (from ~ 1% to ~ 2%) this still shows the functionality of these amylopectin synthesis genes, and how their modification can affect, and ultimately improve these nutritionally important traits. As with any modification in amylopectin biosynthesis pathway, these *ssiia* null mutants had less total starch, and grain width was reduced. Interestingly, the Jag-*ssiia*-∆B and Jag-*ssiia*-∆D mutants showed phenotypic variances that pique an interest and may give insight on the roles of the different homoeologs of *SSIIa* in wheat. Grains of Jag-*ssiia*-∆B had no significant difference in their width, though TGW was still significantly lower than wild type yet significantly higher than the full null mutant. Mutant Jag-*ssiia*-∆D had a seemingly reverse phenotype in amylose content and total starch content, with amylose percentage being significantly lower than the *ssiia* null mutant as well as WT, in addition, and as is the trend, total starch percentage was significantly higher than the *ssiia* null. This study shows the possibility of developing non-GM, nutritionally superior cultivars in an efficient manner. Established, elite cultivars, such as Jagger, are already widely accepted and used by growers, and modifications of single gene for increasing its nutritional value will lead to its easy adoption in production and supply chain as a specialty wheat. This study shows the impact of the modification of one gene in the starch synthesis pathway, and the possibilities of the health benefits that coincide with these modifications.

## Methods

### Development of Jagger TILLING population

Development and characterization of the Jagger TILLING population used in the work has been described in detail in Rawat et al. (2019) [36]. The plant material was procured from Wheat Genetics Resource Center, Kansas State University, Manhattan, Kansas. Development Briefly, 2500 Jagger seeds were treated with a 0.7% EMS solution after a previous dosage optimization step. 1326 M_1_ plants were obtained from the treated (M_0_) and planted in a 1:1 vermiculite/soil mixture. Vernalization was done at the two-leaf stage for 6 weeks at 4 °C. M_1_ Plants were allowed to self, resulting in M_2_ seeds. Out of the 1310 M_2_ seeds planted, 1296 M_2_ plants germinated, each with a unique set of SNPs. M_2_ plants were used for tissue collection, and high throughput DNA extraction was done using Quiagen Biosprint 96 robot with a Biosprint 96 Plant DNA extraction kit (Qiagen) according to the manufacturer’s instruction. DNA quantification was done using a Nanodrop (NanoDrop 200, Thermo Scientific). Dilutions of 25 ng/μL were made in 96-well blocks, and 4X DNA pools were developed by combining 200 μL of normalized DNA from four 96-well blocks, retaining column and row identity. All the plants were grown in greenhouse unto maturity for 16:8 h of day:night length at 22 °C: 18 °C day and night temperatures. The plant research conducted in the work is compliant with institutional, national and international guidelines.

### Identification of *SSIIa* mutants

**Fig. 5:**
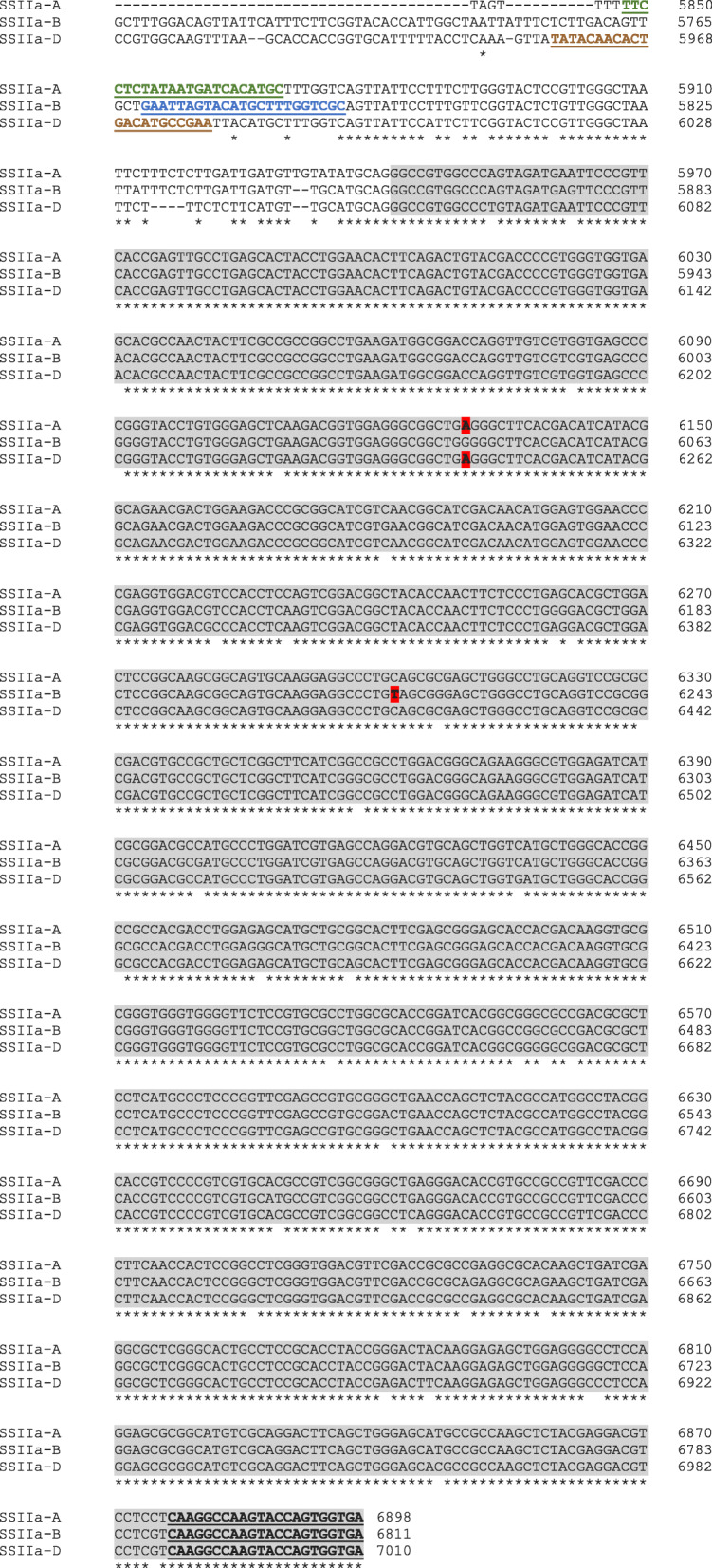
Clustal Omega alignment of Exon 8 (highlighted in grey) and upstream intronic region of the A, B and D homoeoalleles of SSIIa gene in the truncation mutants. The A and D genome common G > A mutation, and the B genome C > T mutation leading to predicted truncation in the three homoeoalleles have been highlighted in red. The position of genome-specific forward primers is depicted in bold and underlined (A genome: green, B genome: blue, D genome: brown), and common reverse primer is bold and underlined in black

Full length gene sequences for the three homoeologous copies of Starch synthase (*SSIIa*) gene were obtained from Shimbata et al. (2005) [48]. Clustal Omega alignments were done, and genome specific primers were designed for all three homoeologous copies (*SSIIa-*A, *SSIIa-*B, *SSIIa-*D) of the *SSIIa* gene keeping the reverse primer common, designed by Shimbata et al. (2005) (Table 1). Figure 5 shows the position and alignment of the primers specific for exon 8 of the three homoeoalleles. Genome specificity was tested using Nullisomic tetrasomic genetic stocks for chromosome 7 from the Wheat Genetics Resource Center, Kansas State University [49]. The targeted genes were amplified separately using the three primer pairs on the 4X TILLING pools first. PCR products were heteroduplexed to form mismatched DNA and home-made *Cel*-1 endonuclease was used to identify mutant pools following the protocol of Rawat et al. (2012) Pools that contained a mutation were then deconvoluted by performing a similar procedure with each individual within the target pool [39]. Two reactions were performed with the individual members of the pool: 1) with only M_2_ DNA and 2) with equal proportions WT Jagger DNA and M_2_ DNA. This allowed for the determination of zygosity of the mutation within the individual. Homozygous mutations appeared only in sample 2 which had WT + mutant DNA, whereas heterozygous mutations appeared as a digested band in both the reactions. Sanger sequencing was done to determine the mutation in the confirmed mutant individuals (Fig. 5). Table 1 shows the number of mutants identified and the knock-out mutants obtained for each genome.

### Development of full null mutant

Plants with knockout mutations in each of the A, B, and D genomes were grown, DNA was extracted, and *Cel*-1 based zygosity determination was done as described above. Individuals with homozygous mutations were selected and crosses were made as A x B, and B x D to generate F1 hybrids with mutations in A-B and B-D homoeoalleles. F1 seeds were harvested and planted. Mutations were confirmed by Sanger sequencing the A-B and B-D homoeoalleles of *SSIIa* in the F1 plants to rule out self-pollination. A double cross was made between these F1 plants to combine all three mutations, retaining the B genome mutation in homozygous condition. Sixty-nine seeds were obtained from the double cross, which were planted again. Zygosity determination was done, and 4 plants were found to have B genome mutation in homozygous condition, and A and D genome mutation in heterozygous condition. These plants were selected and allowed to self. A set of 96 seeds were planted from the bulked selfed seeds, DNA was extracted and zygosity was determined using *Cel*-1 assays. Six plants were found to contain all A, B, and D mutations in homozygous state. Mutations were confirmed by Sanger sequencing in all three genomes. These six plants were allowed to self and seeds were bulked for further analysis of starch composition. Homozygous A, B, and D genome mutants in addition to Wild type Jagger plants were used to compare the parameters of starch composition and grain size comparisons.

### Starch and grain size analysis

One gram of whole dry seeds was manually ground in a mortar pestle to a fine flour for each genotype. Amylose content was determined using Megazyme International’s Amylose/Amylopectin Assay Kit (K-AMYL) as per the manufacturer’s protocol. Six biological reps with two technical reps were preformed with the amylose/amylopectin assay kit. Resistant starch as well as total starch content was determined using Megazyme International’s Resistant Starch Assay Kit (K-RSTAR) as per the manufacturer’s protocol. Three biological reps with two technical reps were preformed with the RS assay kit. Measurements of grain size (*n* = 25) was done using free software ImageJ. After determining homogeneity of variances using Levene Test as well as testing for normality using the Shapiro-Wilk Test, statistical analysis of the results was done using one-way ANOVA, followed by Tukey T-test using R. Correlation analysis was done using the corrplot package in R.

## Compliance with national guidelines on plant research

The plant research conducted in the work is compliant with institutional, national and international guidelines.

## Supplementary Information

**Additional file 1: Supplementary Table 1.** DNA amplicon sequences of wild type Jagger and truncation mutants for SSIIa gene A, B and D genome copies. The red highlighted bases in the mutant sequences in the table indicate the variation of gene sequence in them. **Supplementary Table 2.** Partial protein sequences of wild type Jagger and truncation mutants deduced from the amplicons for SSIIa A, B and D genome copies.

## Data Availability

The sequences of SSIIa amplicons from A, B, and D genomes and their corresponding proteins of wild type Jagger and truncation mutants are available as Supplementary Tables 1 and 2 of this article. These sequences are also available at NCBI as Genbank sequence numbers: MW279062, MW279063, MW279064, MW279065, MW279067, and MW279067. The germplasm developed is available at Wheat Genetics Resource Center, and any correspondence regarding access to the germplasm or DNA and predicted sequences may be addressed to BSG bsgill@ksu.edu or NR nidhirwt@umd.edu.

## Abbreviations

TILLING: Targeting Induced Local Lesions IN Genomes
RDS: Rapidly digestible starches
SDS: Slowly digestible starches
TGW: Thousand grain weight
SSIIa: Starch synthase IIa
SGP-1: Starch granule proteins isoform-1
RS: Resistant starches
SBE: Starch branching enzyme
WT: Wild type parent Jagger
SCFA: Short chain fatty acid
TS: Total starch
