# Supplementary material for: Triple null mutations in starch synthase SSIIa gene homoeologs lead to high amylose and resistant starch in hexaploid wheat
Source: BMC Plant Biol. 2021 Feb 3;21:74. doi: 10.1186/s12870-020-02822-5 (PMC7860177; doi:10.1186/s12870-020-02822-5)
Supplement: Supplementary file 1 — Additional file 1: Supplementary Table 1. DNA amplicon sequences of wild type Jagger and truncation mutants for SSIIa gene A, B and D genome copies. The red highlighted bases in the mutant sequences in the table indicate the variation of gene sequence in them. Supplementary Table 2. Partial protein sequences of wild type Jagger and truncation mutants deduced from the amplicons for SSIIa A, B and D genome copies. [file 12870_2020_2822_MOESM1_ESM.docx]

Supplementary Table 1

DNA amplicon sequences of wild type Jagger and truncation mutants for *SSIIa* gene A, B and D genome copies. The red highlighted bases in the mutant sequences in the table indicate the variation of gene sequence in them.

| Identity of Genotype | DNA amplicon sequence |
| --- | --- |
| A-genome-Jagger-wildtype | TAATGATCACATGCTTTGGTCAGTTATTCCTTTCTTGGGTACTCCGTTGGGCTAATTCTTTCTCTTGATTGATGTTGTATATGCAGGGCCGTGGCCCAGTAGATGAATTCCCGTTCACCGAGTTGCCTGAGCACTACCTGGAACACTTCAGACTGTACGACCCCGTGGGTGGTGAGCACGCCAACTACTTCGCCGCCGGCCTGAAGATGGCGGACCAGGTTGTCGTGGTGAGCCCCGGGTACCTGTGGGAGCTCAAGACGGTGGAGGGCGGCTGGGGGCTTCACGACATCATACGGCAGAACGACTGGAAGACCCGCGGCATCGTCAACGGCATCGACAACATGGAGTGGAACCCCGAGGTGGACGTCCACCTCCAGTCGGACGGCTACACCAACTTCTCCCTGAGCACGCTGGACTCCGGCAAGCGGCAGTGCAAGGAGGCCCTGCAGCGCGAGCTGGGCCTGCAGGTCCGCGCCGACGTGCCGCTGCTCGGCTTCATCGGCCGCCTGGACGGGCAGAAGGGCGTGGAGATCATCGCGGACGCCATGCCCTGGATCGTGAGCCAGGACGTGCAGCTGGTCATGCTGGGCACCGGCCGCCACGACCTGGAGAGCATGCTGCGGCACTTCGAGCGGGAGCACCACGACAAGGTGCGCGGGTGGGTGGGGTTCTCCGTGCGCCTGGCGCACCGGATCACGGCGGGCGCCGACGCGCTCCTCATGCCCTCCCGGTTCGAGCCGTGCGGGCTGAACCAGCTCTACGCCATGGCCTACGGCACCGTCCCCGTCGTGCACGCCGTCGGCGGGCTGAGGGACACCGTGCCGCCGTTCGACCCCTTCAACCACTCCGGCCTCGGGTGGACGTTCGACCGCGCCGAGGCGCACAAGCTGATCGAGGCGCTCGGGCACTGCCTCCGCACCTACCGGGACTACAAGGAGAGCTGGAGGGGCCTCCAGGAGCGCGGCATGTCGCAGGACTTCAGCTGGGAGCATGCCGCCAAGCTCTACGAGGACGTCCTCCTCAAGGCCAAGTACCAGTGGTGA |
| B-genome-Jagger-wildtype | GAATTAGTACATGCTTTGGTCGCAGTTATTCCTTTGTTCGGTACTCTGTTGGGCTAATTATTTCTCTTGATTGATGTTGCATGCAGGGCCGTGGCCCAGTAGATGAGTTCCCGTTCACCGAGTTGCCTGAGCACTACCTGGAACACTTCAGACTGTACGACCCCGTGGGTGGTGAACACGCCAACTACTTCGCCGCCGGCCTGAAGATGGCGGACCAGGTTGTCGTCGTGAGCCCGGGGTACCTGTGGGAGCTGAAGACGGTGGAGGGCGGCTGGGGGCTTCACGACATCATACGGCAGAACGACTGGAAGACCCGCGGCATCGTGAACGGCATCGACAACATGGAGTGGAACCCCGAGGTGGACGTCCACCTCAAGTCGGACGGCTACACCAACTTCTCCCTGGGGACGCTGGACTCCGGCAAGCGGCAGTGCAAGGAGGCCCTGCAGCGGGAGCTGGGCCTGCAGGTCCGCGGCGACGTGCCGCTGCTCGGCTTCATCGGGCGCCTGGACGGGCAGAAGGGCGTGGAGATCATCGCGGACGCGATGCCCTGGATCGTGAGCCAGGACGTGCAGCTGGTCATGCTGGGCACCGGGCGCCACGACCTGGAGGGCATGCTGCGGCACTTCGAGCGGGAGCACCACGACAAGGTGCGCGGGTGGGTGGGGTTCTCCGTGCGGCTGGCGCACCGGATCACGGCCGGCGCCGACGCGCTCCTCATGCCCTCCCGGTTCGAGCCGTGCGGACTGAACCAGCTCTACGCCATGGCCTACGGCACCGTCCCCGTCGTGCATGCCGTCGGCGGCCTGAGGGACACCGTGCCGCCGTTCGACCCCTTCAACCACTCCGGGCTCGGGTGGACGTTCGACCGCGCAGAGGCGCAGAAGCTGATCGAGGCGCTCGGGCACTGCCTCCGCACCTACCGGGACTACAAGGAGAGCTGGAGGGGGCTCCAGGAGCGCGGCATGTCGCAGGACTTCAGCTGGGAGCATGCCGCCAAGCTCTACGAGGACGTCCTCGTCAAGGCCAAGTACCAGTGGTGA |
| D-genome-Jagger-wildtype | TGACATGCCGAATTACATGCTTTGGTCAGTTATTCCATTCTTCGGTACTCCGTTGGGCTAATTCTTTCTCTTCATGTTGCATGCAGGGCCGTGGCCCTGTAGATGAATTCCCGTTCACCGAGTTGCCTGAGCACTACCTGGAACACTTCAGACTGTACGACCCCGTGGGTGGTGAACACGCCAACTACTTCGCCGCCGGCCTGAAGATGGCGGACCAGGTTGTCGTGGTGAGCCCCGGGTACCTGTGGGAGCTGAAGACGGTGGAGGGCGGCTGGGGGCTTCACGACATCATACGGCAGAACGACTGGAAGACCCGCGGCATCGTCAACGGCATCGACAACATGGAGTGGAACCCCGAGGTGGACGCCCACCTCAAGTCGGACGGCTACACCAACTTCTCCCTGAGGACGCTGGACTCCGGCAAGCGGCAGTGCAAGGAGGCCCTGCAGCGCGAGCTGGGCCTGCAGGTCCGCGCCGACGTGCCGCTGCTCGGCTTCATCGGCCGCCTGGACGGGCAGAAGGGCGTGGAGATCATCGCGGACGCCATGCCCTGGATCGTGAGCCAGGACGTGCAGCTGGTGATGCTGGGCACCGGGCGCCACGACCTGGAGAGCATGCTGCAGCACTTCGAGCGGGAGCACCACGACAAGGTGCGCGGGTGGGTGGGGTTCTCCGTGCGCCTGGCGCACCGGATCACGGCGGGGGCGGACGCGCTCCTCATGCCCTCCCGGTTCGAGCCGTGCGGGCTGAACCAGCTCTACGCCATGGCCTACGGCACCGTCCCCGTCGTGCACGCCGTCGGCGGCCTCAGGGACACCGTGCCGCCGTTCGACCCCTTCAACCACTCCGGGCTCGGGTGGACGTTCGACCGCGCCGAGGCGCACAAGCTGATCGAGGCGCTCGGGCACTGCCTCCGCACCTACCGAGACTTCAAGGAGAGCTGGAGGGCCCTCCAGGAGCGCGGCATGTCGCAGGACTTCAGCTGGGAGCACGCCGCCAAGCTCTACGAGGACGTCCTCGTCAAGGCCAAGTACCAGTGGTGA |
| A-genome-SSIIa-truncation mutant | TAATGATCACATGCTTTGGTCAGTTATTCCTTTCTTGGGTACTCCGTTGGGCTAATTCTTTCTCTTGATTGATGTTGTATATGCAGGGCCGTGGCCCAGTAGATGAATTCCCGTTCACCGAGTTGCCTGAGCACTACCTGGAACACTTCAGACTGTACGACCCCGTGGGTGGTGAGCACGCCAACTACTTCGCCGCCGGCCTGAAGATGGCGGACCAGGTTGTCGTGGTGAGCCCCGGGTACCTGTGGGAGCTCAAGACGGTGGAGGGCGGCTGAGGGCTTCACGACATCATACGGCAGAACGACTGGAAGACCCGCGGCATCGTCAACGGCATCGACAACATGGAGTGGAACCCCGAGGTGGACGTCCACCTCCAGTCGGACGGCTACACCAACTTCTCCCTGAGCACGCTGGACTCCGGCAAGCGGCAGTGCAAGGAGGCCCTGCAGCGCGAGCTGGGCCTGCAGGTCCGCGCCGACGTGCCGCTGCTCGGCTTCATCGGCCGCCTGGACGGGCAGAAGGGCGTGGAGATCATCGCGGACGCCATGCCCTGGATCGTGAGCCAGGACGTGCAGCTGGTCATGCTGGGCACCGGCCGCCACGACCTGGAGAGCATGCTGCGGCACTTCGAGCGGGAGCACCACGACAAGGTGCGCGGGTGGGTGGGGTTCTCCGTGCGCCTGGCGCACCGGATCACGGCGGGCGCCGACGCGCTCCTCATGCCCTCCCGGTTCGAGCCGTGCGGGCTGAACCAGCTCTACGCCATGGCCTACGGCACCGTCCCCGTCGTGCACGCCGTCGGCGGGCTGAGGGACACCGTGCCGCCGTTCGACCCCTTCAACCACTCCGGCCTCGGGTGGACGTTCGACCGCGCCGAGGCGCACAAGCTGATCGAGGCGCTCGGGCACTGCCTCCGCACCTACCGGGACTACAAGGAGAGCTGGAGGGGCCTCCAGGAGCGCGGCATGTCGCAGGACTTCAGCTGGGAGCATGCCGCCAAGCTCTACGAGGACGTCCTCCTCAAGGCCAAGTACCAGTGGTGA |
| B-genome-SSIIa-truncation mutant | GAATTAGTACATGCTTTGGTCGCAGTTATTCCTTTGTTCGGTACTCTGTTGGGCTAATTATTTCTCTTGATTGATGTTGCATGCAGGGCCGTGGCCCAGTAGATGAGTTCCCGTTCACCGAGTTGCCTGAGCACTACCTGGAACACTTCAGACTGTACGACCCCGTGGGTGGTGAACACGCCAACTACTTCGCCGCCGGCCTGAAGATGGCGGACCAGGTTGTCGTCGTGAGCCCGGGGTACCTGTGGGAGCTGAAGACGGTGGAGGGCGGCTGGGGGCTTCACGACATCATACGGCAGAACGACTGGAAGACCCGCGGCATCGTGAACGGCATCGACAACATGGAGTGGAACCCCGAGGTGGACGTCCACCTCAAGTCGGACGGCTACACCAACTTCTCCCTGGGGACGCTGGACTCCGGCAAGCGGCAGTGCAAGGAGGCCCTGTAGCGGGAGCTGGGCC  TGCAGGTCCGCGGCGACGTGCCGCTGCTCGGCTTCATCGGGCGCCTGGACGGGCAGAAGGGCGTGGAGATCATCGCGGACGCGATGCCCTGGATCGTGAGCCAGGACGTGCAGCTGGTCATGCTGGGCACCGGGCGCCACGACCTGGAGGGCATGCTGCGGCACTTCGAGCGGGAGCACCACGACAAGGTGCGCGGGTGGGTGGGGTTCTCCGTGCGGCTGGCGCACCGGATCACGGCCGGCGCCGACGCGCTCCTCATGCCCTCCCGGTTCGAGCCGTGCGGACTGAACCAGCTCTACGCCATGGCCTACGGCACCGTCCCCGTCGTGCATGCCGTCGGCGGCCTGAGGGACACCGTGCCGCCGTTCGACCCCTTCAACCACTCCGGGCTCGGGTGGACGTTCGACCGCGCAGAGGCGCAGAAGCTGATCGAGGCGCTCGGGCACTGCCTCCGCACCTACCGGGACTACAAGGAGAGCTGGAGGGGGCTCCAGGAGCGCGGCATGTCGCAGGACTTCAGCTGGGAGCATGCCGCCAAGCTCTACGAGGACGTCCTCGTCAAGGCCAAGTACCAGTGGTGA |
| D-genome-SSIIa-truncation mutant | TGACATGCCGAATTACATGCTTTGGTCAGTTATTCCATTCTTCGGTACTCCGTTGGGCTAATTCTTTCTCTTCATGTTGCATGCAGGGCCGTGGCCCTGTAGATGAATTCCCGTTCACCGAGTTGCCTGAGCACTACCTGGAACACTTCAGACTGTACGACCCCGTGGGTGGTGAACACGCCAACTACTTCGCCGCCGGCCTGAAGATGGCGGACCAGGTTGTCGTGGTGAGCCCCGGGTACCTGTGGGAGCTGAAGACGGTGGAGGGCGGCTGAGGGCTTCACGACATCATACGGCAGAACGACTGGAAGACCCGCGGCATCGTCAACGGCATCGACAACATGGAGTGGAACCCCGAGGTGGACGCCCACCTCAAGTCGGACGGCTACACCAACTTCTCCCTGAGGACGCTGGACTCCGGCAAGCGGCAGTGCAAGGAGGCCCTGCAGCGCGAGCTGGGCCTGCAGGTCCGCGCCGACGTGCCGCTGCTCGGCTTCATCGGCCGCCTGGACGGGCAGAAGGGCGTGGAGATCATCGCGGACGCCATGCCCTGGATCGTGAGCCAGGACGTGCAGCTGGTGATGCTGGGCACCGGGCGCCACGACCTGGAGAGCATGCTGCAGCACTTCGAGCGGGAGCACCACGACAAGGTGCGCGGGTGGGTGGGGTTCTCCGTGCGCCTGGCGCACCGGATCACGGCGGGGGCGGACGCGCTCCTCATGCCCTCCCGGTTCGAGCCGTGCGGGCTGAACCAGCTCTACGCCATGGCCTACGGCACCGTCCCCGTCGTGCACGCCGTCGGCGGCCTCAGGGACACCGTGCCGCCGTTCGACCCCTTCAACCACTCCGGGCTCGGGTGGACGTTCGACCGCGCCGAGGCGCACAAGCTGATCGAGGCGCTCGGGCACTGCCTCCGCACCTACCGAGACTTCAAGGAGAGCTGGAGGGCCCTCCAGGAGCGCGGCATGTCGCAGGACTTCAGCTGGGAGCACGCCGCCAAGCTCTACGAGGACGTCCTCGTCAAGGCCAAGTACCAGTGGTGA |

Supplementary Table 2

Partial protein sequences of wild type Jagger and truncation mutants deduced from the amplicons for SSIIa A, B and D genome copies

| Identity of Genotype | Predicted protein sequence from the amplicons |
| --- | --- |
| A-genome-Jagger-wild type | GRGPVDEFPFTELPEHYLEHFRLYDPVGGEHANYFAAGLKMADQVVVVSPGYLWELKTVEGGWGLHDIIRQNDWKTRGIVNGIDNMEWNPEVDVHLQSDGYTNFSLSTLDSGKRQCKEALQRELGLQVRADVPLLGFIGRLDGQKGVEIIADAMPWIVSQDVQLVMLGTGRHDLESMLRHFEREHHDKVRGWVGFSVRLAHRITAGADALLMPSRFEPCGLNQLYAMAYGTVPVVHAVGGLRDTVPPFDPFNHSGLGWTFDRAEAHKLIEALGHCLRTYRDYKESWRGLQERGMSQDFSWEHAAKLYEDVLLKAKYQW |
| B-genome-SSIIa-Jagger-wild-type | GRGPVDEFPFTELPEHYLEHFRLYDPVGGEHANYFAAGLKMADQVVVVSPGYLWELKTVEGGWGLHDIIRQNDWKTRGIVNGIDNMEWNPEVDVHLKSDGYTNFSLGTLDSGKRQCKEALQRELGLQVRGDVPLLGFIGRLDGQKGVEIIADAMPWIVSQDVQLVMLGTGRHDLEGMLRHFEREHHDKVRGWVGFSVRLAHRITAGADALLMPSRFEPCGLNQLYAMAYGTVPVVHAVGGLRDTVPPFDPFNHSGLGWTFDRAEAQKLIEALGHCLRTYRDYKESWRGLQERGMSQDFSWEHAAKLYEDVLVKAKYQW |
| D-genome-SSIIa-Jagger-wild-type | GRGPVDEFPFTELPEHYLEHFRLYDPVGGEHANYFAAGLKMADQVVVVSPGYLWELKTVEGGWGLHDIIRQNDWKTRGIVNGIDNMEWNPEVDAHLKSDGYTNFSLRTLDSGKRQCKEALQRELGLQVRADVPLLGFIGRLDGQKGVEIIADAMPWIVSQDVQLVMLGTGRHDLESMLQHFEREHHDKVRGWVGFSVRLAHRITAGADALLMPSRFEPCGLNQLYAMAYGTVPVVHAVGGLRDTVPPFDPFNHSGLGWTFDRAEAHKLIEALGHCLRTYRDFKESWRALQERGMSQDFSWEHAAKLYEDVLVKAKYQW |
| A-genome-SSIIa-truncation mutant | GRGPVDEFPFTELPEHYLEHFRLYDPVGGEHANYFAAGLKMADQVVVVSPGYLWELKTVEGG |
| B-genome-SSIIa-truncation mutant | GRGPVDEFPFTELPEHYLEHFRLYDPVGGEHANYFAAGLKMADQVVVVSPGYLWELKTVEGGWGLHDIIRQNDWKTRGIVNGIDNMEWNPEVDVHLKSDGYTNFSLGTLDSGKRQCKEAL |
| D-genome-SSIIa-truncation mutant | GRGPVDEFPFTELPEHYLEHFRLYDPVGGEHANYFAAGLKMADQVVVVSPGYLWELKTVEGG |
